# Multitargeted Approach for the Optimization of Morphogenesis and Barrier Formation in Human Skin Equivalents

**DOI:** 10.3390/ijms22115790

**Published:** 2021-05-28

**Authors:** Arnout Mieremet, Richard W. J. Helder, Andreea Nadaban, Walter A. Boiten, Gert S. Gooris, Abdoelwaheb El Ghalbzouri, Joke A. Bouwstra

**Affiliations:** 1 Department of Dermatology, Leiden University Medical Center, 2333 ZA Leiden, The Netherlands; a.mieremet@lumc.nl (A.M.); a.e.l.ghalbzouri@lumc.nl (A.E.G.); 2 Division of BioTherapeutics, Leiden Academic Centre of Drug Research, Leiden University, 2333 CD Leiden, The Netherlands; r.w.j.helder@lacdr.leidenuniv.nl (R.W.J.H.); a.nadaban@lacdr.leidenuniv.nl (A.N.); w.a.boiten.2@lacdr.leidenuniv.nl (W.A.B.); gooris_g@lacdr.leidenuniv.nl (G.S.G.)

**Keywords:** human skin equivalents, skin, ceramides, free fatty acids, monounsaturated, nuclear receptors/LXR, chitosan, hypoxia

## Abstract

In vitro skin tissue engineering is challenging due to the manifold differences between the in vivo and in vitro conditions. Yet, three-dimensional (3D) human skin equivalents (HSEs) are able to mimic native human skin in many fundamental aspects. However, the epidermal lipid barrier formation, which is essential for the functionality of the skin barrier, remains compromised. Recently, HSEs with an improved lipid barrier formation were generated by (i) incorporating chitosan in the dermal collagen matrix, (ii) reducing the external oxygen level to 3%, and (iii) inhibiting the liver X receptor (LXR). In this study, we aimed to determine the synergic effects in full-thickness models (FTMs) with combinations of these factors as single-, double-, and triple-targeted optimization approaches. The collagen–chitosan FTM supplemented with the LXR inhibitor showed improved epidermal morphogenesis, an enhanced lipid composition, and a better lipid organization. Importantly, barrier functionality was improved in the corresponding approach. In conclusion, our leading optimization approach substantially improved the epidermal morphogenesis, barrier formation, and functionality in the FTM, which therefore better resembled native human skin.

## 1. Introduction

In-vitro-developed human skin equivalents (HSEs) replicate multiple critical aspects of in vivo skin biology due to the presence of a nourishing culture medium and a supportive micro-environment [1,2,3]. Numerous types of three-dimensional (3D) HSEs exist, ranging from epidermal models to skin-on-a-chip models [4,5,6,7]. Nevertheless, a shared restriction in all models is the reduced barrier formation [8,9,10,11]. This affects the resemblance to the native human skin (NHS) tissue and limits the applicability in pharmacokinetics and toxicological screenings [12]. Therefore, there is an urgent need to improve epidermal morphogenesis and barrier formation in HSEs.

The stratum corneum (SC) is the main layer of the physical epidermal barrier [13]. This outermost epidermal layer is composed of corneocytes that are embedded in a lipid matrix. The corneocytes contain a cornified envelope consisting of highly cross-linked proteins, resulting in almost impermeable objects [13]. The lipid matrix is the only continuous pathway through the SC, which is crucial for the functionality of the skin barrier [14,15]. Within the lipid matrix, the lipids are organized in lipid lamellae. Two major repetitive lamellar phases can be distinguished, which are termed the short periodicity phase (SPP) and the long periodicity phase (LPP) [16,17]. Perpendicular to the plane of the lamellae, the lipids are organized in lateral packing. From very dense to less dense, these lipids can adopt an orthorhombic, hexagonal, or liquid lateral organization [16,17]. Principally, the lipid organization is a result of the lipid composition. The main SC lipid classes are ceramides (CERs), free fatty acids (FFAs), and cholesterol [15]. The CERs contribute 50% by mass to the lipid composition and are a key player in the formation of structured lamellae [14,15]. CERs vary in head group architecture, resulting in the presence of at least 16 subclasses [18,19,20]. Furthermore, both CERs and FFAs can differ in the number of carbons in the acyl chains and the degree of unsaturation, yielding a high diversity of lipid entities within the SC matrix. Variations in the lipid composition can induce changes in the lipid organization and functionality [21,22,23].

The lipid barrier formation is usually characterized based on the expression of lipid biosynthesis enzymes, lipid composition, lipid organization, and barrier functionality. Characterization of the lipid barrier formation in HSEs revealed similar features as compared to that of NHS. These similarities include the biosynthesis of all lipid classes, the extrusion of lamellar bodies at the stratum granulosum (SG)–SC interface, the presence of all CER subclasses with a wide distribution in carbon chain length, and the formation of organized lipid lamellae anchored to the lipid envelope [8,24]. Differences were observed in the lipid compositions. These cover the altered CER subclass profile, the increased presence of CERs with a low number of carbon atoms (<40 carbon atoms), and a higher level of monounsaturated CERs (muCERs) and monounsaturated FFAs (muFFAs). This is linked to an elevated expression of the lipid desaturase enzyme stearoyl-CoA desaturase-1 (SCD-1) in HSEs [8,24,25,26].

As the in vitro barrier formation of HSEs is a result of complex interactions, it is currently not fully understood. Therefore, we investigated the barrier formation of HSEs via an innovative multitargeted strategy using our in-house-developed HSE, which is referred to as the full-thickness model (FTM). The targets were identified based on results obtained in previous studies, which aimed to optimize single aspects of 3D skin tissue engineering, which are the extracellular matrix (ECM) composition, external culture conditions, and the medium composition [27,28,29]. These studies demonstrated that (i) the addition of chitosan to the ECM, (ii) the reduction of external culture conditions to hypoxic levels, and (iii) the addition of the liver X receptor (LXR) antagonist GSK2033 all led to improvements in the FTMs.

In this study, we aimed to evaluate the synergic effects of improved dermal ECM, external factors, and medium composition on epidermal morphogenesis and lipid barrier formation in HSEs (Figure S1a–g). Therefore, FTMs and collagen–chitosan FTMs (CC-FTMs) were generated under normoxic or hypoxic conditions with or without LXR inhibition. Herein, we report that our leading optimization approaches resulted in well-structured epidermal morphogenesis with enhanced barrier formation, contributing to improved barrier functionality in FTMs.

## 2. Results

To determine whether there was a cumulative effect for the different optimization approaches, single and combined approaches were tested, such as the addition of chitosan to the dermal compartment, the supplementation of the LXR antagonist GSK2033, or the reduction of oxygen. These conditions are referred to as single-, double-, and triple-targeted approaches and are described in Table 1.

In the following paragraphs, the different FTM characteristics, such as the epidermal morphogenesis, lipid composition, and lipid organization created by these different approaches, are presented.

### 2.1. Multitargeted Optimization Approach Improved Epidermal Morphogenesis

**Figure 1:**
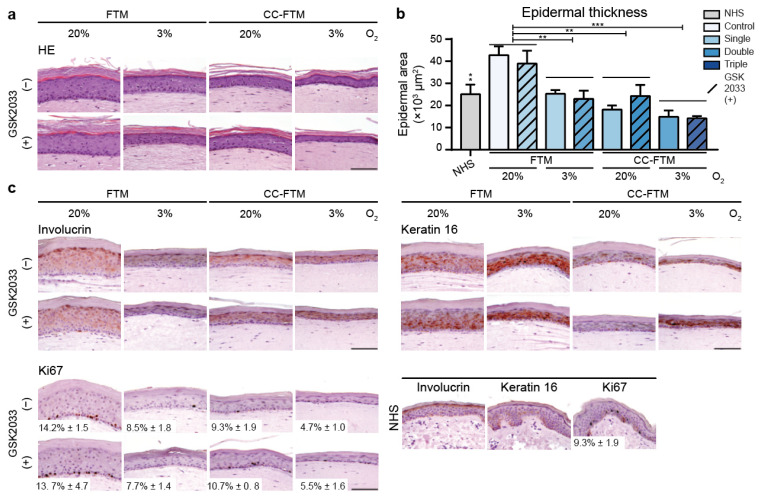
Epidermal morphogenesis of single-, double-, and triple-targeted FTMs. (**a**) Histology of FTMs generated with single-, double-, and triple-targeted approaches. Single targets were the dermal matrix composition (FTM or CC-FTM), oxygen level (20% or 3%, FTM(3) and FTM(20), respectively), or inhibition of LXR by 500 nM GSK2033 (FTM(−)). Double- and triple-targeted FTMs were generated using a combination of these. (**b**) Thickness of the viable epidermis of NHS and the indicated FTMs. (**c**) Epidermal morphogenesis was examined via the expression of protein biomarkers for late differentiation (involucrin), activation (keratin 16), and proliferation (Ki67). Representative images are shown with biomarkers in red and nuclei in blue, where the scale bar indicates 100 μm. All data are represented as mean + sd, *n* = 3. Significant differences are indicated by ** for *p* < 0.01 and *** for *p* < 0.001.

The single-, double-, and triple-targeted FTMs were evaluated for their histological appearance, which revealed a well-orchestrated epidermal stratification in all conditions (Figure 1a). However, in some approaches, a reduction in thickness of the viable epidermis was observed as compared to the control FTM(20−) (Figure 1b). FTM(3−), FTM(3+), CC-FTM(20−), and CC-FTM(20+) displayed the highest resemblance to the epidermal thickness of NHS. Furthermore, the shape of granular cells was more flattened in these four models. The evaluation of epidermal morphogenesis using protein biomarkers confirmed the late differentiation using involucrin expression (Figure 1c). FTM(3−), FTM(3+), CC-FTM(20−), and CC-FTM(20+) most closely mimicked the late differentiation program of NHS, although involucrin remained overexpressed in the stratum spinosum in all FTMs. Epidermal activation was determined via keratin 16 (K16) expression, which was expressed in the control FTM(20−) in contrast to no expression in NHS. Although K16 remained expressed in the majority of FTMs, K16 expression was reduced in CC-FTM(20−) and CC-FTM(20+), thereby better resembling the epidermal activation state of NHS. Proliferation was evaluated via Ki67 expression, which was restricted to the basal cell layer in all conditions. However, a high proliferation index was observed in FTM(20−) and FTM(20+), which was reduced in hypoxia and the CC-FTMs, with additional reduction after the combination of these in CC-FTM(3−) and CC-FTM(3+). Together, these results indicate that the epidermal morphogenesis was improved when using our optimization strategy, which was mainly induced by the modification of the dermal ECM by chitosan.

### 2.2. Multitargeted Optimization Approach in FTMs Led to Enhanced Ceramide Composition

**Figure 2:**
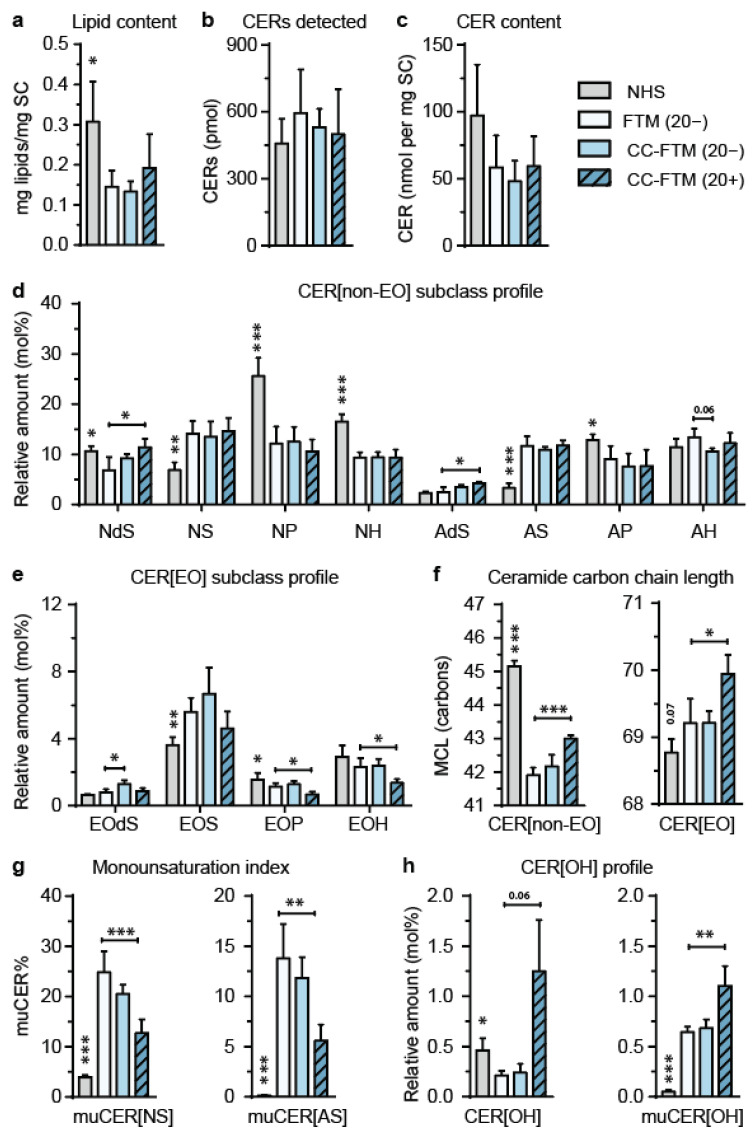
Ceramide composition profile in the leading optimization approaches. (**a**) Total lipids in the SC of a selection of single- and double-targeted FTMs. (**b**) The level of total detected CERs that were found using LC–MS in a 1500 ng lipid extract of NHS and indicated FTMs. (**c**) Absolute amount of CER in the SC as shown per milligram of the SC of NHS and indicated FTMs. (**d**) CER[non-EO] subclass profile in NHS and indicated FTMs, shown in relative amounts. Data are shown for saturated CERs and are indicated as mean + sd, *n* ≥ 4. (**e**) CER[EO] subclass profile in NHS and indicated FTMs, shown in relative amounts. Data are shown for saturated CERs and are indicated as mean + sd, *n* ≥ 4. (**f**) Mean carbon chain length of CER[non-EO] and CER[EO] in indicated FTMs. (**g**) Level of monounsaturated CERs for subclasses [AS] and [NS]. The level of monounsaturation is indicated by the percentage of muCERs within each subclass. (**h**) The relative amount of saturated and monounsaturated CER[OH]. Data are shown for NHS and single- and double-targeted FTMs. Data are represented as mean + sd, *n* ≥ 4. Significant differences are indicated by * for *p* < 0.05, ** for *p* < 0.01, and *** for *p* < 0.001.

Examination of the barrier formation in FTMs was initiated via the characterization of the SC lipid composition. To present the results efficiently, we show data for NHS, the control FTM(20−), and the FTMs that exhibited the most promising results regarding epidermal morphogenesis and lipid composition. These leading optimization approaches were CC-FTM(20−) and CC-FTM(20+). Nonetheless, complete CER composition datasets are included in Figures S2–S6. Within the SC of selected FTMs, a comparable level of total lipids was detected, although this was lower as compared to that of NHS (Figure 2a). The composition of CERs was examined for the most prevalent CER subclasses [19] (Figure S1g). Liquid chromatography coupled to mass spectrometry (LC–MS) ion maps indicated deviations in the CER composition of FTMs compared to NHS, including the presence of CERs with an *m/z* below 600 (Figure S7).

The data were quantified to determine the CER composition in more detail. This revealed a similar level of detected CERs within the extracted lipids that were derived from FTMs and NHS (Figure 2b). Quantification into absolute amounts per milligram of the SC also revealed that the level of CERs in all FTMs was comparable to NHS (Figure 2c). CER[non-EO] and CER[EO] subclass profiles were plotted in relative amounts as a percentage of the total quantified amount of CERs. The relative amounts were provided as these are directly related to the lipid phase interactions within the lipid organization. Comparisons of the CER[non-EO] subclass profiles between FTMs showed no differences, except for increased amounts of CER[NdS] and [AdS] for FTM(20+) compared to CC-FTM(20−) (Figure 2d). However, as compared to NHS in the CER[non-EO] subclass profile differences were detected for the relative amount of subclasses [NdS], [NS], [NP], [NH], [AS], and [AP]. The CER[EO] subclass profiles showed minor but significant changes between FTMs for [EOdS], [EOP], and [EOH] (Figure 2e). As compared to NHS, the CER[EO] subclass profile revealed substantial differences in the relative amounts of subclasses [EOS] and [EOP].

The absolute amounts of each CER[non-EO] and CER[EO] subclass per milligram of the SC are provided in Figure S4 for all single-, double-, and triple-targeted FTMs. This quantitative data did not show major differences between the various FTMs, including the leading approaches. As compared to NHS, in the FTMs, there was a deficiency in the absolute amounts of [NP], [NH], [EOP], and [EOH], whereas there was an overabundance in the absolute amounts of subclass [AS], again indicating an imbalanced sphingolipid biosynthesis in the FTM as compared to that of NHS.

We next addressed the effects of the targeting approaches on the CER carbon chain length. Therefore, the mean carbon chain lengths (MCLs) of CER[non-EO] and CER[EO] were determined (Figure 2f). A drastic reduction in the MCL of CER[non-EO] was observed in FTM(20−) as compared to that of NHS. Excitingly, the MCL of CER[non-EO] in CC-FTM(20+) was increased as a result of the inhibition of LXR, thereby better mimicking that of NHS. A comparable MCL of CER[EO] in NHS, FTM(20−), and CC-FTM(20−) was observed, while the MCL of CER[EO] in CC-FTM(20+) was increased. To further evaluate the carbon chain length of CER[non-EO] and CER[EO], a distribution profile was plotted for the relative and absolute amounts of CERs per number of carbons (Figure S8). The carbon chain length distributions in the CER[non-EO] and CER[EO] in the various FTMs deviated substantially from that of NHS. In the control FTM(20−), a relative and absolute increased amount of CER[non-EO] ≤ 40 carbon atoms and a reduced amount of CER[non-EO] ≥ 46 carbon atoms was observed. This contributed to the considerably lower MCL in FTM(20−) as compared to NHS. Interestingly, the relative carbon chain length distribution profile of CC-FTM(20+) better resembled that of NHS due to a lower level CER[non-EO] ≤ 40 carbon atoms and a higher level of CER[non-EO] ≥ 46 carbon atoms. This explains the higher MCL of CER[non-EO] in CC-FTM(20+).

Next, the level of muCERs was determined, which was indicated via the percentage of muCERs within the subclasses [NS] and [AS] (Figure 2g). This provided an indication for the fraction of muCERs for all subclasses. In FTMs, the level of muCERs was substantially increased as compared to that in NHS. The percentage of muCERs was significantly reduced after the inhibition of LXR in all conditions (Figure S5), better resembling the level of muCERs in the CER[non-EO] composition of NHS.

The level of muCERs in the CER[EO] composition of the SC was also determined, although due to a higher number of subgroups within the CER[EO] subclasses, this was more complex. Four different subgroups could be distinguished for CER[EO] based on the degree of unsaturation (saCER[EO] or muCER[EO]) and the type of ester-linked FFA residue (oleic acid [EO-18:1] or linoleic acid [EO-18:2]), as illustrated in Figure S1g. These four subgroups were analyzed for the subclass [EOS] in NHS, FTM(20−), CC-FTM(20−), and CC-FTM(20+) (Figure S6). However, saCER[EOS-18:2] and muCER[EOS-18:1] eluted at the same retention time and shared the same *m/z*. Therefore, these are presented as a combined subgroup. When comparing the CER[EOS] subgroup profiles of NHS and control FTM(20−), a major difference was observed in the presence of saCER[EO-18:1]. Strikingly, this subgroup was substantially present in FTMs, whereas it was absent in NHS. This contributed to the increased relative amount of subclass CER[EOS] in the lipid matrix of FTMs as compared to NHS. Comparing the CER[EOS] subgroup profile between the various FTMs, the relative amount of the subgroup of muCER[EOS-18:2] was increased in both CC-FTM(3−) and CC-FTM(3+), but only the combined peak saCER[EOS-18:2] + muCER[EOS-18:1] was increased in the CC-FTM(3−) as compared to control FTM(20−). This indicates only a small difference between the subgroup profiles for CER[EOS] induced by the various optimization approaches.

The lipidomics analysis also included the detection of the ω-hydroxyceramide (CER[O]) subclass. In-depth profiling of this subclass was performed in a recent study by Helder et al. [28], where it was reported that the CER[OH] subclass serves as an indicator for the presence and composition of all CER[O] subclasses. Therefore, the CER[OH] profile was determined as a percentage of the total quantified amount of ceramides in NHS, FTM(20−), CC-FTM(20−), and CC-FTM(20+) (Figure 2h). In NHS, saCER[OH] was the most abundant subclass that was detected. In the various FTMs, saCER[OH] was detected as well, but higher amounts of muCER[OH] were observed. This contributed to an overall increase in the CER[OH] subclass in the FTMs compared to NHS. When comparing the optimization approaches, the addition of the LXR antagonist significantly increased the amount of CER[OH] and muCER[OH] in the lipid matrix of the SC.

In summary, we showed a substantially improved ceramide composition in the double-targeting approaches, which involved LXR inhibition as compared to the control FTM(20−).

### 2.3. Leading Optimization Approaches Improved Organization of the Lipid Matrix

**Figure 3:**
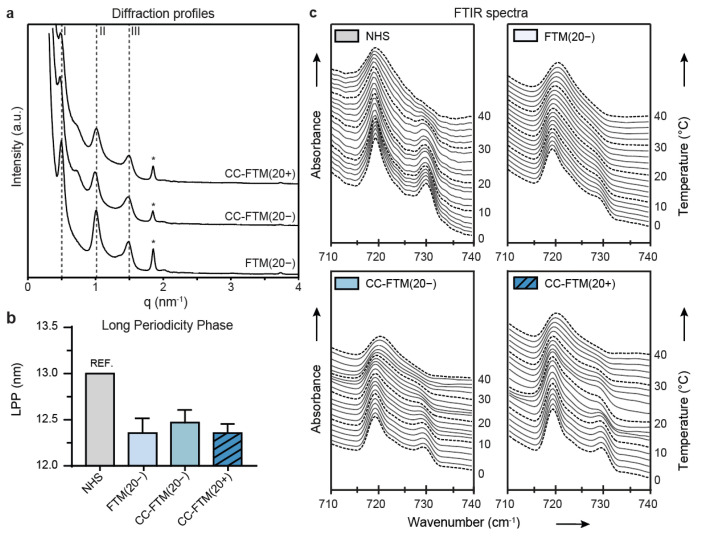
Lipid matrix organization in the SC of NHS and the leading targeted FTMs. (**a**) Small-angle X-ray diffraction profiles of selected single- and multitargeted FTMs. Data are shown as intensity in arbitrary units (a.u.) on the y-axis and scattering vector q on the x-axis. The position of the diffraction orders (I, II, and III) are indicated by dashed lines, whereas the cholesterol phase is indicated by the asterisk symbol (*). (**b**) Bar plot of the calculated repeat distance of the LPP in FTMs. Values for NHS were obtained from Bouwstra et al. [30], data are presented as mean + sd (**c**) FTIR spectra of NHS and selected single- and multitargeted FTMs. Data are presented as absorbance (in a.u.) on the left y-axis over the wavenumber (in cm^−1^) on the x-axis in the absorbance region corresponding to the methylene rocking vibrational mode. On the right y-axis, the temperature measurement range is shown (from 0 to 40 °C).

Complementary to the compositional analyses, we evaluated the lipid organization in the SC of the FTMs that were developed with the leading optimization approaches, which were CC-FTM(20−) and CC-FTM(20+), and NHS and the control FTM(20−). In all FTMs, the lipids assembled in lipid lamellae with repeat distances that are characteristic for the LPP, as detected in the small-angle X-ray diffraction (SAXD) profiles (Figure 3a). From the position of the peaks, the repeat distance of the LPP was calculated, which was similar for all conditions tested (Figure 3b). Nevertheless, the repeat distance in all FTMs was shorter than the reported LPP repeat distance of NHS. Additionally, the SPP was not present in the FTMs, although this lamellar phase is present in NHS [30].

The lateral lipid organization was examined using Fourier transformed infrared (FTIR) spectroscopy in a temperature range between 0 and 40 °C. The spectrum of the methylene rocking vibrational mode was indicative of the orthorhombic lateral packing when there were two peaks present at 719 cm^−1^ and 730 cm^−1^, whereas the hexagonal lateral packing was indicated by a single peak at 719 cm^−1^. In FTM(20−) at 10 °C, the spectra showed a single peak at 719 cm^−1^, indicating that the lipids were predominantly arranged in the hexagonal lateral packing (Figure 3c). In CC-FTM(20−) and CC-FTM(20+), the peak at 730 cm^−1^ was stronger, indicating that a higher fraction of lipids adopted the orthorhombic lateral packing. Yet, in NHS, the peak at 730 cm^−1^ was the most intense, indicating that in NHS, a higher fraction of lipids was predominantly arranged in the orthorhombic lateral packing.

In summary, our analyses showed that the lipid organization in the control FTM(20−) differed from that of NHS, which was partially restored in the leading optimization approaches (CC-FTM) regarding the lipid lateral packing.

### 2.4. Optimized FTMs Displayed Enhanced Barrier Functionality

Evaluation of the barrier formation in the leading targeted FTMs was finalized via the determination of the barrier functionality.

**Figure 4:**
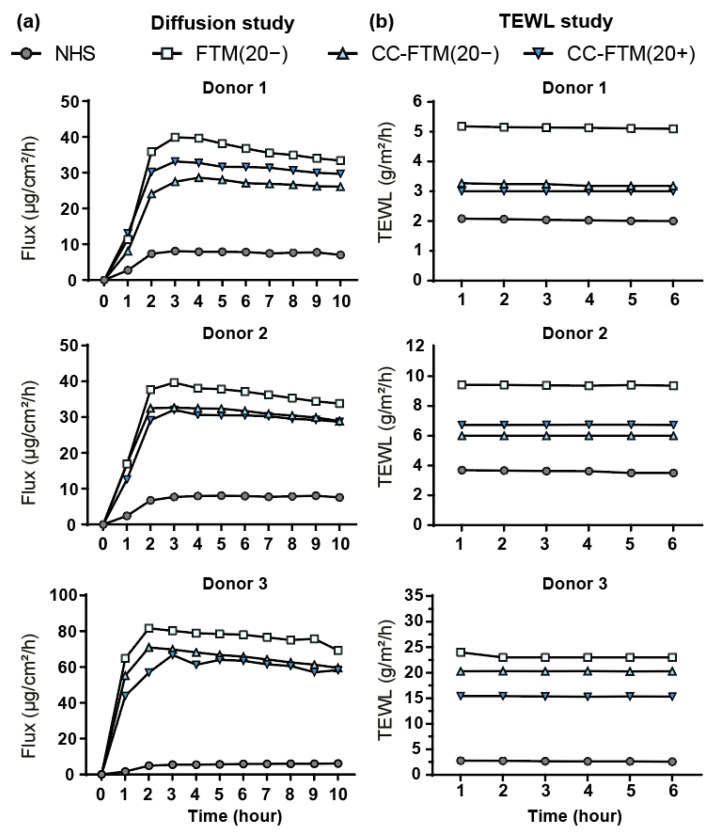
Individual barrier functionality in optimized FTMs. (**a**) Evaluation of the individual outside–in barrier functionality of NHS and selected FTMs over a period of 10 h. (**b**) Evaluation of individual inside–out barrier functionality of NHS and indicated FTMs. Transepidermal water loss (TEWL) was measured over a period of 45 min. The last 5 min are plotted.

For the outside–in barrier functionality assessment, benzocaine flux values were measured for three donors over a period of 10 h (Figure 4a). A steady state for the different donors was observed after three hours. Flux values were lower for the lead optimization approaches as compared to the control FTM(20−) (Figure 4a). Nevertheless, the flux values remained much higher in all FTMs as compared to NHS. Next, the inside–out barrier functionality was examined by measuring the transepidermal water loss (TEWL) over a period of 45 min (Figure 4b). This also revealed an impaired barrier functionality in the control FTM(20−) as compared to NHS. Importantly, the TEWL values were substantially lower in CC-FTM(20−) and CC-FTM(20+). These assessments provided evidence for an enhanced barrier functionality in the lead optimization approaches.

## 3. Discussion

In this study, the optimization of in-vitro-developed FTMs using advanced single-, double-, and triple-targeted approaches resulted in a better resemblance of our FTMs to NHS. This concerned the epidermal morphogenesis after modification of the dermal ECM by chitosan and the SC lipid composition after inhibition of the liver X receptor. These effects persisted by targeting both factors, resulting in improved lipid organization and functionality of the epidermal barrier and closer resemblance to NHS.

The presence of the four epidermal layers of NHS was mirrored in all FTMs, in agreement with results obtained in previous studies [27,28]. In addition, the epidermal morphogenesis of CC-FTM(20−) and CC-FTM(20+) most closely mimicked that of NHS. This was based on the reduced thickness of the viable epidermis, reduced thickness of granular cells, and lower epidermal activation as compared to FTM(20−). The morphogenesis of the double-targeted CC-FTM(3−) and triple-targeted CC-FTM(3+) showed a substantially thinner viable epidermis and lower basal cell proliferation as compared to NHS. This was a result of the combinatory anti-proliferative effects of chitosan, which allowed keratinocytes to better adhere to the basement membrane and the lower oxygen level, which reduced the cellular metabolism rate [31,32,33]. This demonstrates that a combination of promising single targets could lead to suboptimal outcomes.

The CER composition was characterized for all single-, double-, and triple-targeted approaches. The results clearly demonstrated that the inhibition of LXR increased the MCL of CER[non-EO] and reduced the level of monounsaturated CER[AS] and [NS], which is in line with previously obtained findings [28]. Based on previous results, a reduction in monounsaturated subclasses belonging to CER[non-EO] was therefore expected as well [28]. These effects were observed irrespective of the dermal equivalent type or external oxygen level, indicating the robustness of the LXR inhibition via GSK2033 supplementation of the culture medium. Unfortunately, the alterations in the CER composition induced by the dermal equivalent type (FTM or CC-FTM) or external oxygen levels that were observed in earlier studies [27,29] did not fully persist in this study. This might be explained by donor variability and/or batch-to-batch variations.

The lipid organization in the SC of FTM was improved after the leading optimized conditions were used. The lamellar phases were comparable, while the lateral organization was improved in the leading approaches as compared to the control FTM(20−). The fraction of lipids that adopted an orthorhombic packing was increased in both CC-FTM(20−) and CC-FTM(20+). Although CC-FTM(20+) had further reduced levels of muCERs, the lateral organization was similar compared to FTM(20−). This implicates that in CC-FTM, other factors besides reduced monounsaturation could contribute to the formation of the orthorhombic lateral packing, such as a possible increased ratio of FFA to CER [28,34,35,36]. Future studies should aim to optimize the ratio of CER/FFA/cholesterol in FTMs, which could promote the formation of the orthorhombic lateral packing.

A detailed lipid comparison between the FTMs and NHS also revealed an increase in CER[non-EO] with ≤40 carbon atoms in all FTMs. It was shown that CER[non-EO] with 34 carbon atoms as compared to CER[non-EO] with 42 carbon atoms in lipid membrane models modified the lipid organization and reduced the barrier functionality [37]. Therefore a reduction in CER[non-EO] with ≤40 carbon atoms in FTMs is an important target to improve lipid organization and barrier functionality. Furthermore, the increased presence of CER[EO-18:1] in the lipid matrix also contributes to an altered barrier formation [38]. The increased presence of CER[EO-18:1] was also observed in essential fatty-acid-deficient skin [39,40]. Therefore, the amount of supplemented linoleic acid (C18:2) is ripe for optimization, thereby potentially reducing the presence of CER[EO-18:1] and favoring the presence of CER[EO-18:2], leading to improvements in the barrier formation of FTMs. However, the incorporation of linoleic acid into triglycerides or other complex lipids instead of into CER[EO] remains a limiting factor [41,42].

Next, the increased presence of CER[O] in the lipid matrix after LXR inhibition was much higher than in NHS. In NHS, CER[EO] is processed via several mechanisms to bind to the corneocyte lipid envelope. During these steps, linoleic acid is removed from the CER[EO] in order to covalently bind CER[O] to the cornified envelope [43]. However, free CER[O] is present in the lipid matrix in both NHS and FTMs [43]. Previously, we examined all enzymes involved in the binding of the CER to the cornified envelope and did not observe any indication that the expression of these enzymes changed after LXR inhibition [28,43,44,45,46]. Furthermore, we observed that the amount of CERs that were bound to the cornified envelope did not change upon LXR inhibition, indicating that the increased amount of CER[O] was not due to a change in the cornified envelope bound lipids. Therefore, it remains to be elucidated why saturated and monounsaturated CER[O] are more abundantly present in the lipid matrix after LXR inhibition.

In this study, barrier function was assessed for the leading optimization approaches through outside–in and inside–out functionality evaluations. The diffusion study was performed with benzocaine, which has a low molecular weight (165.189 g/mol) and has medium lipophilicity (logP = 1.9). It serves as a model compound and is also recommended by the OECD guidelines [47,48,49]. As the organization of lipids showed an increased fraction of lipids adopting the orthorhombic packing, this was accurately reflected by lower benzocaine diffusion fluxes in the corresponding CC-FTMs. Similar results were observed for the inside–out permeability, where the diffusion of water through the SC was monitored, showing that both CC-FTMs had lower TEWL values [50]. Although the lipid composition changes in the CC-FTM(20−) were less consistent in this study, the decreased TEWL values that were measured remained persistent [27]. In addition, previous studies performed with the LXR antagonist displayed improvement in the lipid composition, as well as the lateral lipid organization, but did not result in a change in TEWL values [28]. Another observation was that, in this study, the level of lipids adopting an orthorhombic packing remained visible even at 24 °C in both CC-FTM(20−) and CC-FTM(20+), which was not observed in the LXR study [28]. Considering, the difference in lipid composition between CC-FTM(20−) and CC-FTM(20+) but a similar lateral organization was observed in both CC-FTMs, this demonstrates that the improved inside–out barrier functionality is not fully explained by the measured lipid profiles. It remains possible that the formation of the corneocyte envelope was enhanced, which led to reduced water evaporation through the SC [51]. Moreover, a clear association of percutaneous penetration with the TEWL in the currently developed in vitro FTMs is more difficult to ascertain; this is also observed in different models, such as full-thickness pig skin [52], in contrast to an established correlation found in NHS [53,54].

In conclusion, FTMs developed with advanced optimization approaches showed substantial improvements for epidermal morphogenesis in combination with enhanced lipid barrier formation and SC functionality, thereby more closely mimicking NHS.

## 4. Materials and Methods

### 4.1. Generation of Human Skin Equivalents

Isolation and cell culturing of primary cells isolated from the dermis and epidermis were performed as described before [55]. The culture medium of all isolated primary cells was tested for mycoplasma contamination by qPCR and was found to be negative. FTMs and CC-FTMs were generated as described before [8,27,28,29,56]. In short, four different batches of FTMs were developed using different primary cell donors. The extracellular matrix containing the fibroblasts was cultured in 14 days. Subsequently, the keratinocytes were seeded and were cultured for another 14 days at the air–liquid interface. For the barrier functionality assays, an additional batch of FTMs was generated with the lead conditions. This was performed due to the limited availability of primary cells and the high sample demand of these barrier functionality assays.

In addition, the following provides a brief explanation regarding the FTM conditions: (i) an optimal chitosan:collagen ratio of 3:1 *v*/*v* was used, as established in a previous study [27]. (ii) Reduction to hypoxic conditions (3% O_2_) was performed in a Heracell™ 240 CO_2_ incubator (ThermoFisher, Waltham, MT, United States). (iii) Supplementation with 500 nM LXR inhibitor GSK2033 (Merck KGaA, Darmstadt, Germany) occurred twice a week, four times in total during the air-exposed phase. Furthermore, treatment with a vehicle (dimethyl sulfoxide) to dissolve the LXR antagonist (0.05% *w*/*v* of the medium) did not induce additional effects for all performed analyses and was therefore excluded from the results.

### 4.2. NHS Used in this Study

NHS was used for cell isolation to generate the FTMs, as described in Section 4.1. In addition, before being used for cell isolation, small samples were taken upon arrival of the skin (at day 0). This was performed to produce a reference value for the FTM comparison. No culturing was performed and the samples taken for the different analysis techniques were processed similarly to those described in the corresponding section.

### 4.3. Immunohistochemical Analyses

Tissue fixation occurred in 4% formaldehyde for 24 h before rehydration and paraffin embedment. Tissue was cut with a 5 μm thickness for hematoxylin and eosin (HE) staining and immunohistochemical analyses. HE staining was performed according to instructions provided by the manufacturer (VWR, Breda, The Netherlands). Immunohistochemical analyses were performed as described before using the following antibodies: Ki67 (1:100, clone MIB, DAKO, Glostrup, Denmark), involucrin (1:1200, clone SY5, Sanbio, Uden, The Netherlands), and keratin 16 (1:100, clone LL025, Serotec, Kidlington, UK) [27]. Estimations of the epidermal thickness were performed on at least six regions per sample according to methods reported before [27]. The proliferation index was determined on two different central regions per sample by counting the Ki67 positive cells in a region with at least 100 basal cells.

### 4.4. Liquid Chromatography Coupled to Mass Spectrometry Analyses

Stratum corneum was isolated using trypsin digestion steps and stored until use in an inert environment, as described before [57]. Lipids were extracted using a modified Bligh and Dyer extraction procedure, as reported before [19,58]. The dry weight of the SC was determined before and after lipid extraction using a microbalance. Extracted lipids were evaporated and reconstituted in an appropriate volume of heptane/chloroform/methanol (95/2.5/2.5 *v*/*v*/*v*) to obtain a 0.3 mg/mL concentration. Ceramide N(24deuterated)S(18) (Evonik Industries, Essen, Germany) was added as the internal standard (ISTD). Liquid chromatography coupled to mass spectrometry (LC–MS) analyses were performed as described by Boiten et al. [19]. In brief, a 5 µL sample volume was injected into the ultra-pressure liquid chromatograph (UPLC) (Waters, Milford, MA, USA) equipped with a PVA-Sil column (5 µm particles size, 100 × 2.1 mm i.d., YMC, Kyoto, Japan). Detection occurred via a XEVO TQ-S mass spectrometer (Waters), measuring full scan *m*/*z* between 350 and 1200 atomic mass units. Data were analyzed with Masslynx software (Masslynx 4.1, Waters, Milford, MA, USA), corrected for ISTD, and quantified using a 3D response model according to methods as described before [19].

### 4.5. SAXD

SAXD measurements were performed at the European Synchrotron Radiation Facility (ESRF, Grenoble, France) at station BM26B for a period of 2 × 90 s, as described elsewhere [21]. The scattering intensity I was measured as a function of the scattering vector *q*. The latter is defined as $q=\frac{4\cdot \;\pi \cdot \sin\theta }{\lambda }$, in which *θ* is the scattering angle and *λ* is the wavelength of the X-rays. From the positions of the peaks, the repeat distance of a lamellar phase was calculated using the equation $d=\frac{2n\cdot \;\pi }{qn}$, where *n* is the order of the diffraction peak of this phase and *qn* is the position of the *n*-order peak.

### 4.6. FTIR Spectroscopy

The isolated SC was rehydrated for 12 h in a desiccator containing deuterated H_2_O. After rehydration, the SC was placed between two AgBr windows prior to measurements. Spectra were acquired on a Varian 670-IR spectrometer (Varian, Palo Alto, CA, USA) equipped with a broadband mercury cadmium telluride detector. The Fourier transform infrared spectroscopy (FTIR) spectrometer was connected to a customized temperature regulator to facilitate measurements at temperatures ranging from 0 to 40 °C. The spectrometer collected the data with a frequency range of 400–4000 cm^−1^ with a spectral resolution of 1 cm^−1^. Measurements were performed in 240 s segments during a total run of 164 min. Varian Resolution Pro software 4.1 (Varian, Palo Alto, CA, USA) was used to analyze and deconvolute the FTIR spectra.

### 4.7. Barrier Functionality Assays

The permeation study was performed using isolated SC placed on a supporting dialysis membrane (Diachema, Munich, Germany) that was mounted into a Permegear inline diffusion cell (Bethlehem, PA, USA) using settings that were described before [21,49]. The donor compartment consisted of a saturated solution of benzocaine (165.189 g/mol) in an acetate buffer (pH 5.0). The acceptor compartment consisted of phosphate-buffered saline solution (pH 7.4), which was stirred and perfused at a flow rate of about 2 mL/h. The diffusing area between the donor and acceptor compartment was 0.282 cm^2^. Each experiment was performed under occlusive conditions at a temperature of 32 °C. Analysis of the amount of diffused benzocaine was performed using a UPLC system. The flux of benzocaine through each sample was calculated using Fick’s first law of diffusion, as described earlier [21]. Measurements of the transepidermal water loss (TEWL) were performed at controlled ambient conditions. The isolated SC was supported by a dialysis membrane and placed in the diffusion cell, which was filled with Millipore water. After a 15 min equilibration period, the closed chamber evaporimeter (Aqua Flux AF200, Biox Systems Ltd., London, UK) was connected with an adapter to the apical side of the diffusion cell for 30 min. Subsequently, measurements were performed every 20 s for at least 6 min [27].

### 4.8. Statistics

Statistical analyses were conducted using GraphPad Prism version 7.0 for Windows (GraphPad Software, La Jolla, CA, USA). Statistical testing was performed between single- and multitargeted FTMs using one-way or two-way ANOVA with Holm–Sidak post-tests. The NHS versus the control FTM, referred to as FTM(20−), for comparison was statistically tested using the unpaired Student’s t-test. Statistical differences are noted with lines for the former and vertically above NHS for the latter comparison, unless otherwise indicated. Significance is indicated by *, **, or *** corresponding to *p* < 0.05, <0.01, or <0.001, respectively.

### 4.9. Data Availability

All data generated or analyzed for this study is included in this published article and its supplementary files.

## Figures and Tables

**Table 1:** Overview of the single-, double-, and triple-targeted optimization approaches: type of FTM (either the control or chitosan), oxygen levels were either normal (20%) or hypoxic (3%), and supplementation of GSK2033 is denoted as (+).

Number	HSE Type	O_2_	GSK2033	Treatment	Abbreviation
1	FTM	20%	(−)	Control	FTM(20−)
2	FTM	20%	(+)	Single	FTM(20+)
3	FTM	3%	(−)	Single	FTM(3−)
4	FTM	3%	(+)	Double	FTM(3+)
5	CC-FTM	20%	(−)	Single	CC-FTM(20−)
6	CC-FTM	20%	(+)	Double	CC-FTM(20+)
7	CC-FTM	3%	(−)	Double	CC-FTM(3−)
8	CC-FTM	3%	(+)	Triple	CC-FTM(3+)

## Data Availability

Data is contained within the article or supplementary material. However, datasets are available on request due to restrictions, PhD candidate requires (part of) the research sets, since they contain multiple studies.

## Supplementary Materials

The following are available online at https://www.mdpi.com/article/10.3390/ijms22115790/s1.
