# Supplementary material for: Multitargeted Approach for the Optimization of Morphogenesis and Barrier Formation in Human Skin Equivalents
Source: Int J Mol Sci. 2021 May 28;22(11):5790. doi: 10.3390/ijms22115790 (PMC8198964; doi:10.3390/ijms22115790)
Supplement: Supplementary file 1 [file ijms-22-05790-s001.zip › ijms-1200461-SI.pdf]

Article

Multitargeted Approach for the Optimization of Morphogenesis and Barrier Formation in Human Skin Equivalents

Arnout Mieremet [1,†], Richard W. J. Helder [2,†], Andreea Nadaban [2], Walter A. Boiten [2], Gert S. Gooris [2], Abdoelwaheb El Ghalbzouri [1,‡] and Joke A. Bouwstra [2,‡,*]

[1] Department of Dermatology, Leiden University Medical Center, 2333 ZA Leiden, The Netherlands; a.mieremet@lumc.nl (A.M.); a.e.l.ghalbzouri@lumc.nl (A.E.G.)
[2] Division of BioTherapeutics, Leiden Academic Centre of Drug Research, Leiden University, 2333 CD Leiden, The Netherlands; r.w.j.helder@lacdr.leidenuniv.nl (R.W.J.H.); a.nadaban@lacdr.leidenuniv.nl (A.N.); w.a.boiten.2@lacdr.leidenuniv.nl (W.A.B.); gooris_g@lacdr.leidenuniv.nl (G.S.G.)
* Correspondence: bouwstra@lacdr.leidenuniv.nl; Tel.: +31-71-527-4208
† First authors contributed equally to this work.
‡ Last Authors contributed equally to this work

Supplementary material

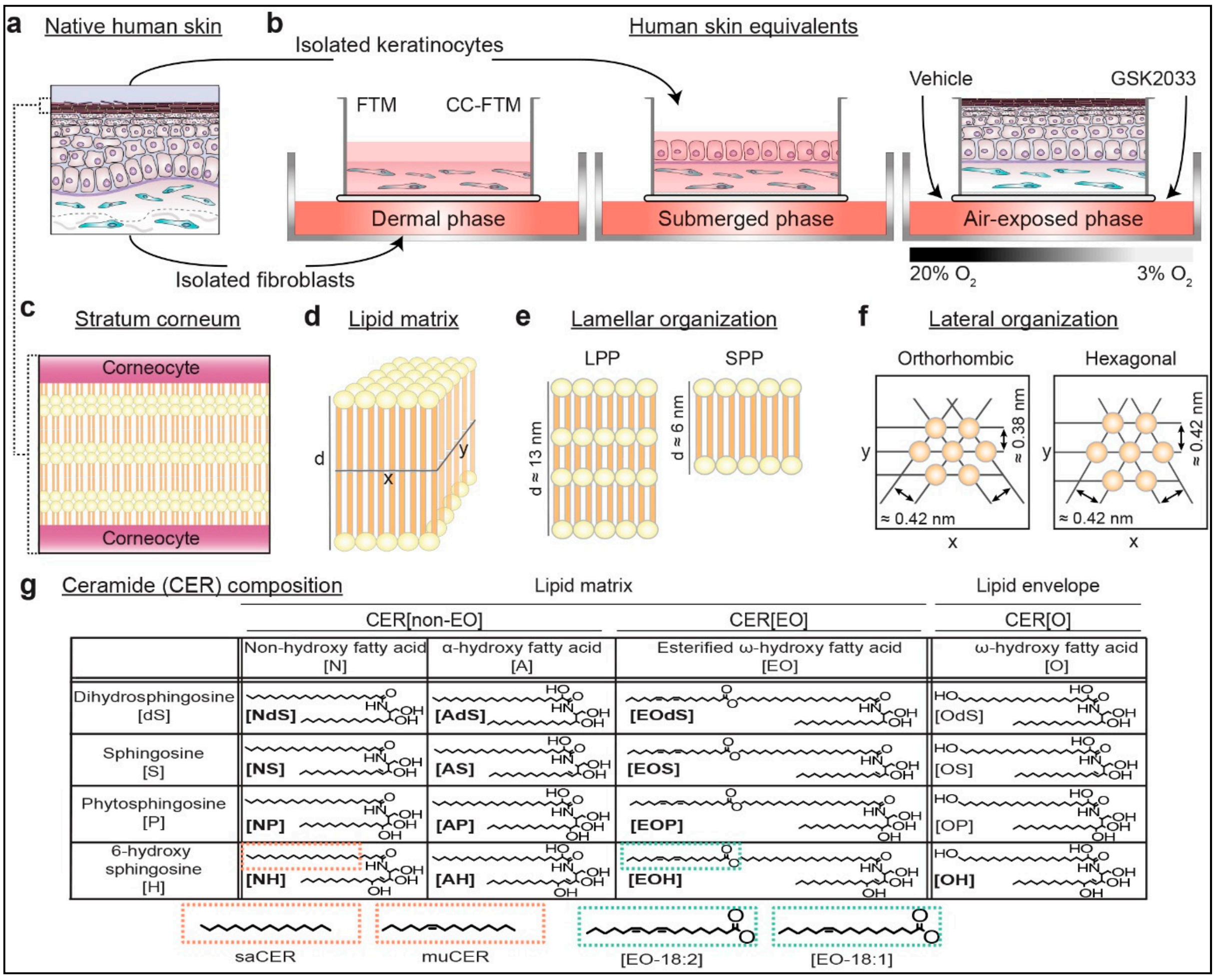

Figure S1. Illustration of study approach and key concepts of the lipid barrier. (a) Schematic overview of a cross section of the native human skin showing the dermis containing fibroblasts and the epidermis containing keratinocytes. (b) Study approach for the development of single- and multitargeted FTMs. Development of FTMs occurred in three main phases. 1) Dermal phase: isolated primary fibroblasts were seeded in a collagen or in a collagen-chitosan extracellular matrix, to develop FTMs or CC-FTMs respectively. 2) Submerged phase: isolated primary keratinocytes were seeded on top of the dermal equivalent. 3) Air-exposed phase: Differentiation occurred leading to full epidermal stratification. In this study,

external oxygen level was reduced to 3% and/or LXR antagonist GSK2033 was added to the medium (c) Schematic overview of the intercorneocyte lipid matrix of the stratum corneum. (d) Illustration of the lipid matrix. Hydrophilic head group regions and hydrophobic hydrocarbon chain regions were indicated by the yellow dots or orange columns respectively. (e) Simplified overview of the lamellar organization. Long periodicity phase (LPP) and short periodicity phase (SPP) as shown with characteristic repeat distance. (f) Lateral organization of the hydrocarbon chains. The orthorhombic organization is a very dense ordered packing, whereas the hexagonal organization is a dense ordered packing. (g) Ceramide composition with subclasses indicated and named after their combination of sphingosine base and free fatty acid chain, according to Motta et al. [1] and expanded as reviewed by Vávrová et al. [2]. Additional CER[non-EO] entities exist based on their saturated CER (saCER) or monounsaturated CER (muCER) acyl-chains, whereas additional CER[EO] entities exist based on the esterified linoleic (CER[EO-18:2]) or oleic (CER[EO-18:1]) acid residue. In this study, the ceramide subclasses indicated in bold were analyzed.

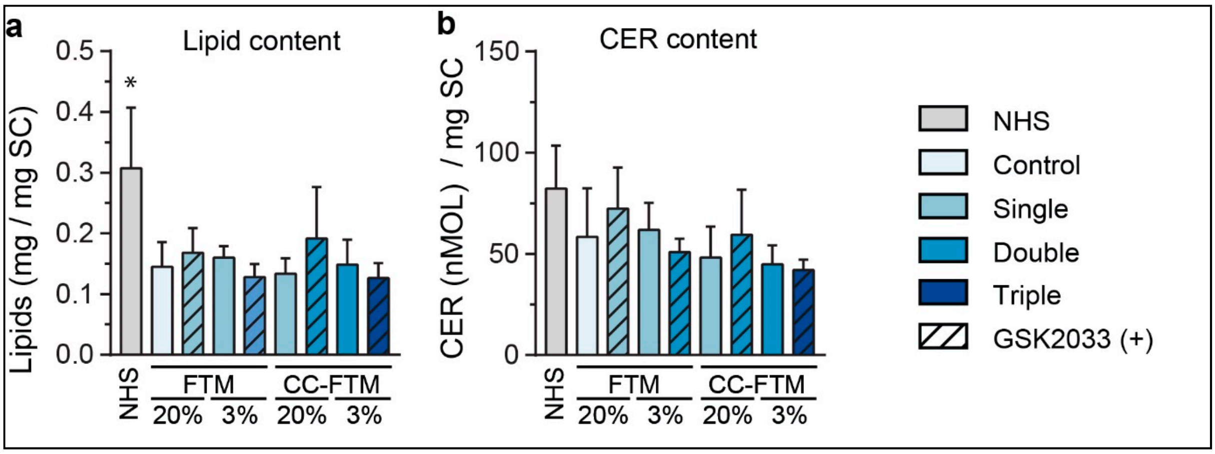

Figure S2. Total lipid and ceramide content in the SC of all single, double, and triple targeted FTMs. (a) Total lipids in the SC of NHS and all single- and multitargeted FTMs and NHS. (b) Absolute amount of CER in the SC as shown per mg SC of NHS and of all single- and multitargeted FTMs. Data represents mean + sd, n≥4 Significant differences are indicated by * for $P_{<0.05.}$

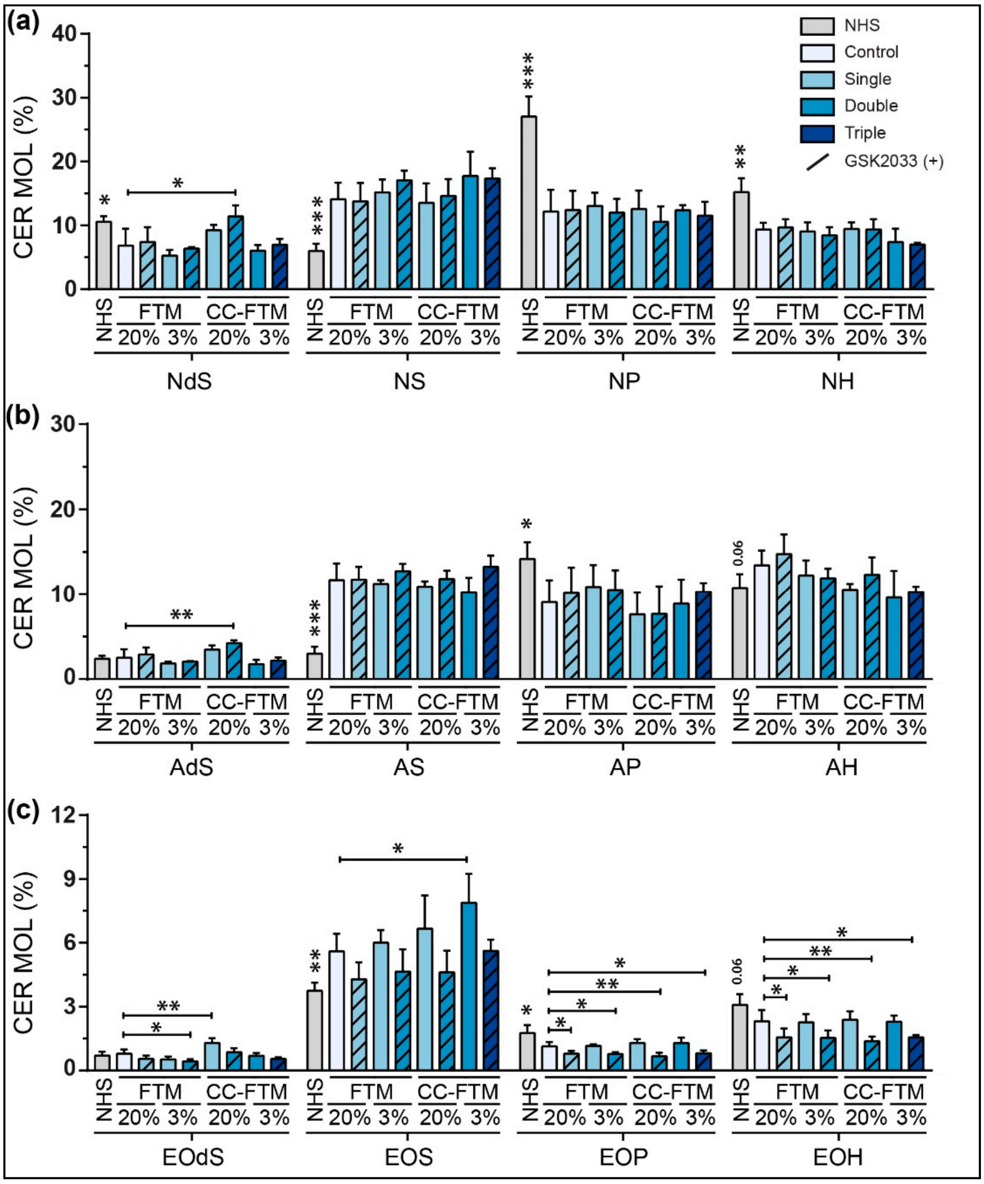

Figure S3. Ceramide subclass profiles of all single, double, and triple targeted FTMs. Relative amount of (a) non-hydroxy CER[non-EO], (b) alpha-hydroxy CER[non-EO], and (c) esterified omega-hydroxy CER[EO] in NHS and FTMs. Data represents saturated CERs and is presented in relative amounts as mean + sd, n≥4. Significant differences are indicated by * for $P_{<0.05}$, ** for $P_{<0.01}$, and *** for $P_{<0.001}$.

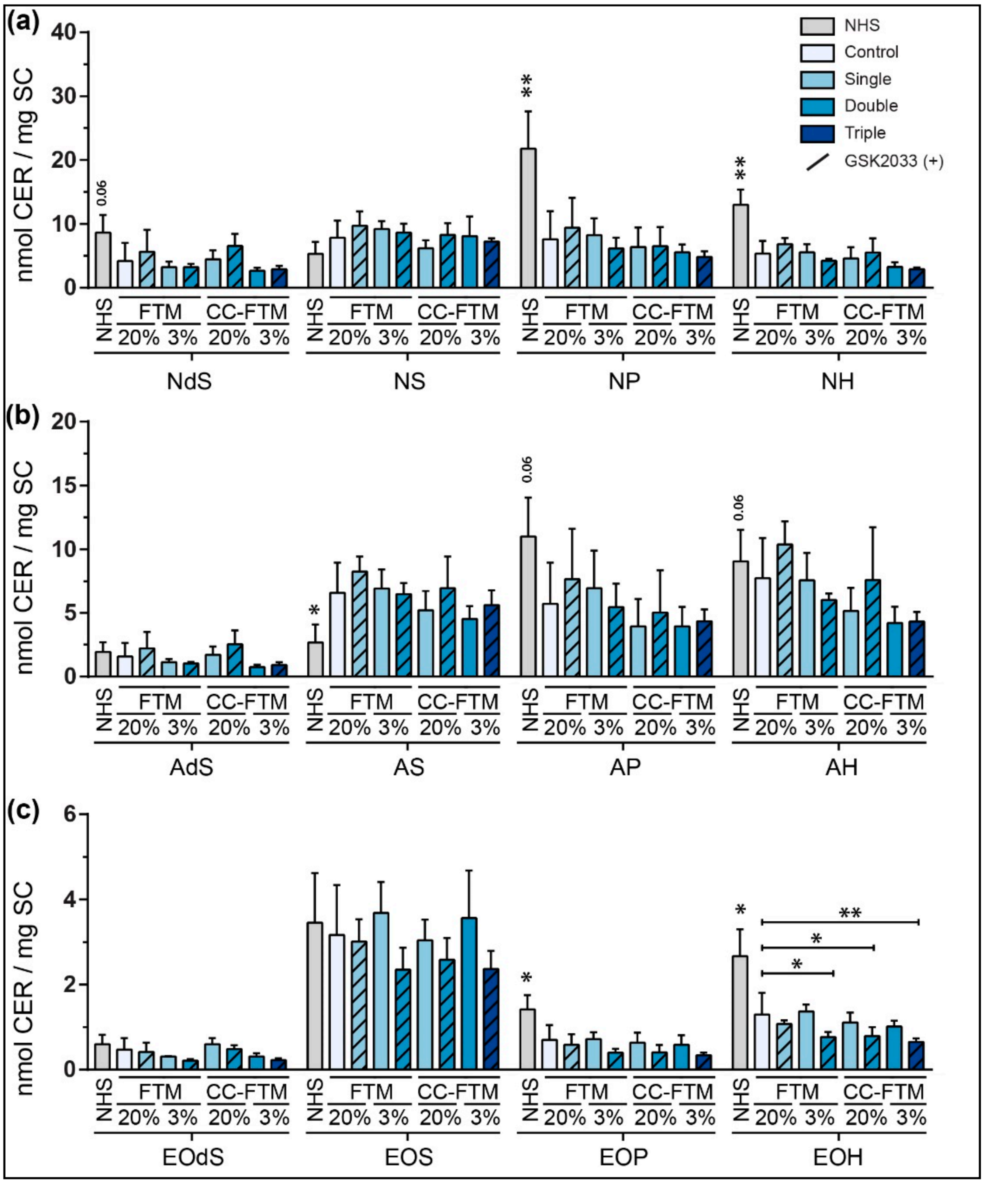

Figure S4. Quantitative ceramide subclass profiles of all single, double, and triple targeted FTMs. Absolute amount of (a) non-hydroxy CER[non-EO], (b) alpha-hydroxy CER[non-EO], and (c) esterified omega-hydroxy CER[EO] in NHS and FTMs. Data represents saturated CERs and is presented in absolute amounts per mg SC as mean + sd, n≥4. Significant differences are indicated by * for $P_{<0.05}$, ** for $P_{<0.01}$.

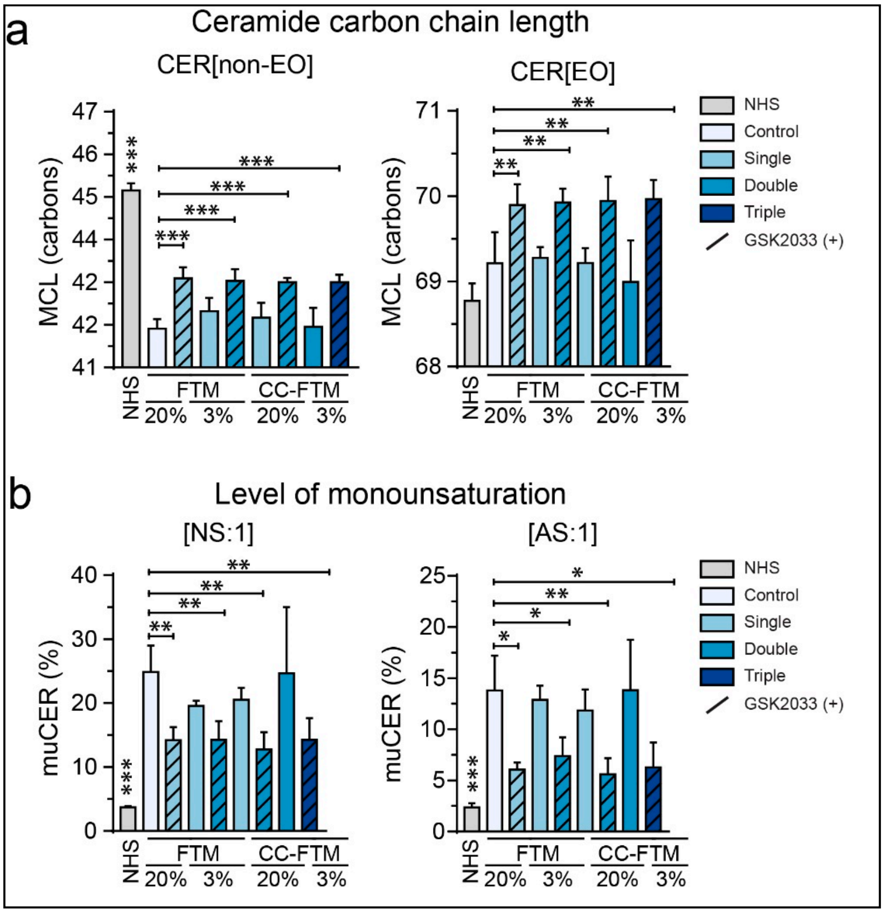

Figure S5. Mean ceramide carbon chain lengths and level of monounsaturation in all single, double, and triple targeted FTMs. (a) Mean carbon chain length of CER[non-EO] and of CER[EO]. (b) Level of monounsaturation presented as percentage of monounsaturated ceramide per subclass. The muCER[NS] and muCER[AS] are shown, which contain a single desaturation in their acyl chain. Data represents mean + sd, n≥4. Significant differences are indicated by * for $P_{<0.05}$, ** for $P_{<0.01}$, and *** for $P_{<0.001}$.

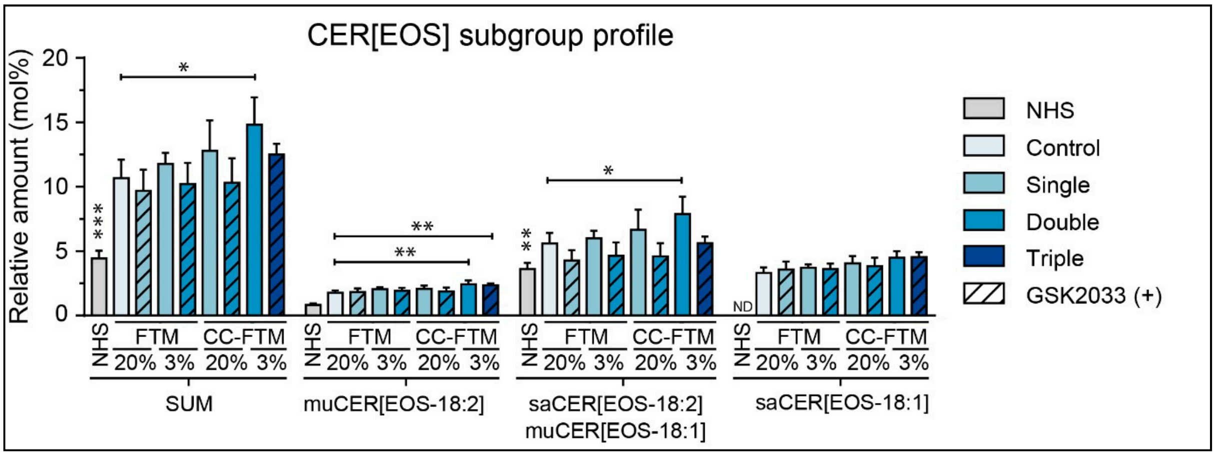

Figure S6. Subgroup profile of subclass EOS in all single, double and triple targeted FTMs. Subgroups are created based on level of desaturation (saCER and muCER and on type of esterified FFA residue (linoleic acid (18:2) or oleic acid (18:1)). This results in four subgroups for each CER[EO] subclass: muCER[EOS-18:2], muCER[EOS-18:1], saCER[EOS-18:2], and saCER[EOS-18:1]. No distinction can be made in detection between muCER[EOS-18:1] and saCER[EOS-18:2], therefore these are indicated as mixed subgroup. Relative amount of subclass CER[EOS] is presented as sum of all and per subgroup. Data represents percentage of the total quantified amount as mean + sd, n≥4. ND indicates no detection. Significant differences are indicated by * for $P_{<0.05,}$ *and* ** for $P_{<0.01.}$

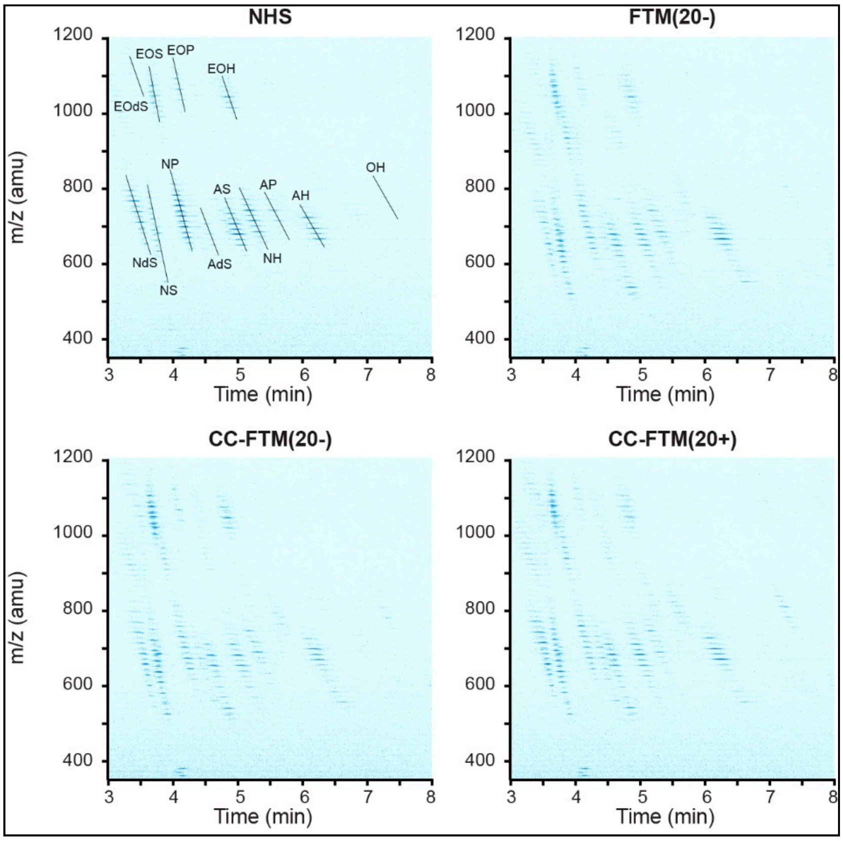

Figure S7. LC-MS ion maps of detected ceramides in lead optimization approaches. Two dimensional LC-MS ion maps with indicated position and nomenclature of the 13 studied CER subclasses in the ion map of NHS.

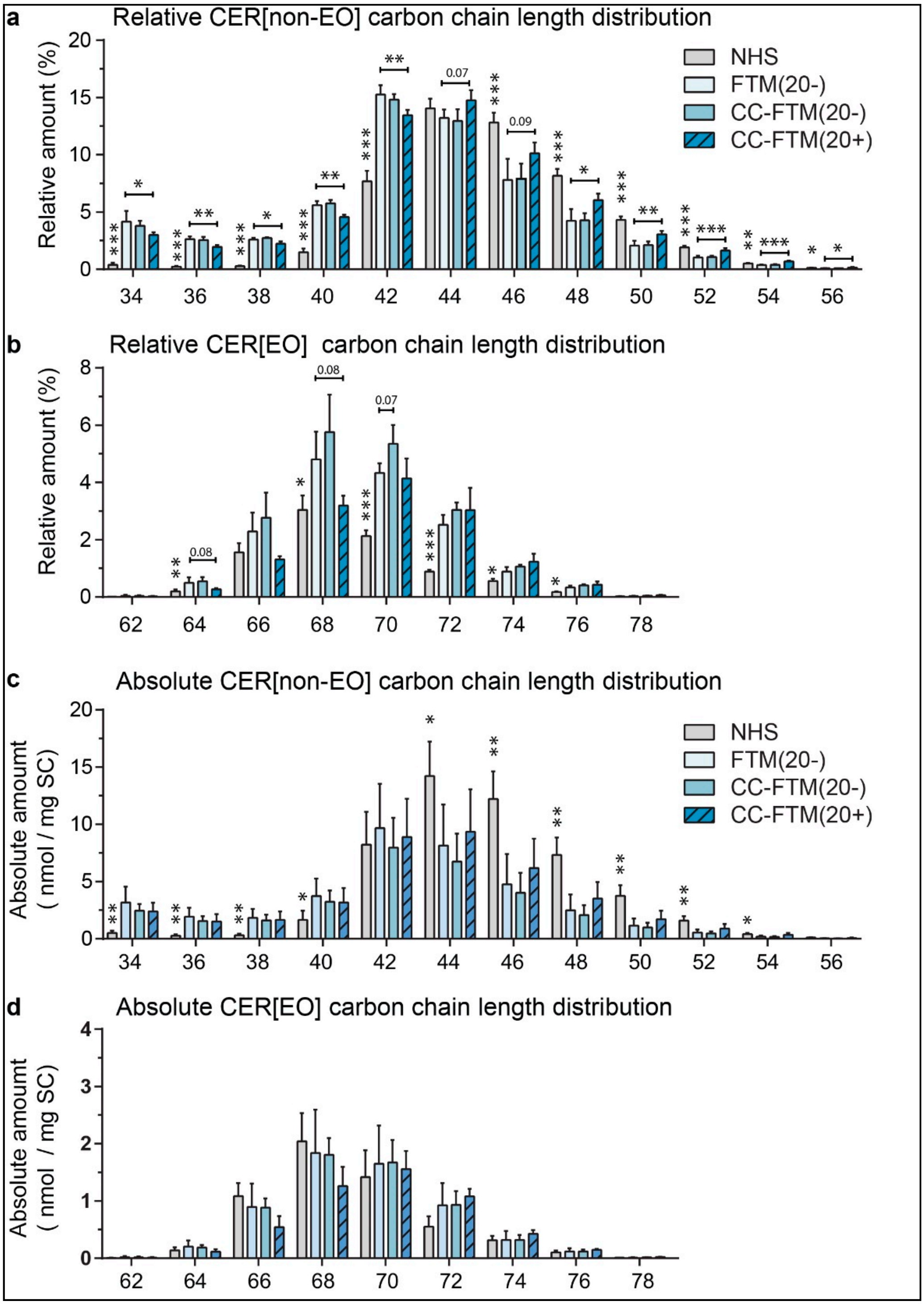

Figure S8. Ceramide carbon chain length distribution of lead optimization approaches. (a) Relative amount each ceramide with a specific amount of carbon atoms for CER[non-EO] and for (b) CER[EO] in NHS and in selected FTMs. (c) Quantitative amount each ceramide with a specific amount of carbon atoms for CER[non-EO] and for (d) CER[EO] in NHS and in selected FTMs. Data represents saturated CERs values in absolute amounts per mg SC as mean + sd, n≥4. Significant differences are indicated by * for $P_{<0.05}$, ** for $P_{<0.01}$, and *** for $P_{<0.001}$.

References

1. Motta, S., et al., *Ceramide composition of the psoriatic scale.* Biochimica et Biophysica Acta - Molecular Basis of Disease, 1993. **1182**(2): p. 147-151.
2. Vávrová, K., A. Kováčik, and L. Opálka, *Ceramides in the skin barrier.* Acta Facultatis Pharmaceuticae Universitatis Comenianae, 2017. **64**(2): p. 28-35.
